# Involvement of MIR-126 and MMP9 in the Pathogenesis of Intra-Abdominal Fistulizing Crohn’s Disease: A Brief Research Report

**DOI:** 10.3389/fsurg.2022.822407

**Published:** 2022-05-10

**Authors:** Cristina Luceri, Mario D’Ambrosio, Elisabetta Bigagli, Lorenzo Cinci, Edda Russo, Fabio Staderini, Marta Cricchio, Francesco Giudici, Stefano Scaringi

**Affiliations:** ^1^ Department of Neuroscience, Psychology, Drug Research and Child Health - NEUROFARBA, Section of Pharmacology and Toxicology, University of Florence, Florence, Italy; ^2^ Department of Surgery and Translational Medicine, Section of Surgery, University of Florence, Florence, Italy; ^3^ Surgical Unit, Careggi Teaching Hospital, Florence, Italy

**Keywords:** Crohn’s disease, miRNA, intra-abdominal fistulas, MMP9, surgery

## Abstract

**Background:**

Intra-abdominal fistulas are complications that affect a significant proportion of Crohn’s disease patients, often requiring surgery. The aim of the present work was to correlate the occurrence of intestinal fistulization to the clinico-pathological features of these patients and to the plasma levels of MMP9, a gelatinase involved in the pathophysiology of fistula formation, and of miR-126, appearing to modulate MMP9 expression.

**Methods:**

In a series of 31 consecutive Crohn’s patients admitted to surgery due to therapeutic failure and/or complicated disease, we identified nine cases of abdominal fistulas, mainly entero-enteric fistulas. MMP9 protein was determined in plasma and at the intestinal level using immunometric assays. Circulating miR-126 was also measured in all plasma samples by real-time PCR.

**Results:**

Comparing patients with and without intra-abdominal fistulas, we did not observe differences in terms of age, gender, disease location and duration, number of previous surgeries and pre-biologic medications. However, cases with intra-abdominal fistulas had a significantly higher CDAI (*p* < 0.0001) and a significantly lower circulating miR-126 (*p* < 0.05). Patients with intra-abdominal fistulas had also a significantly higher amount of circulating MMP9 (*p* < 0.0001) and this data was correlated with an increased expression of MMP9 protein in the mucosa and with reduced levels of circulating miR-126. Receiver operating characteristic (ROC) analysis pointed out the ability of circulating MMP9 to discriminate patients with and without intra-abdominal fistulas.

**Conclusions:**

These data confirm that circulating MMP9 can be used for the identification of cases with intra-abdominal fistulas and suggest that miR-126 may be also involved in the pathogenesis of this complication and that it may be further investigated as a new therapeutic strategy or for monitoring therapeutic response in these patients.

## Introduction

The development of fistulas in the course of Crohn’s disease (CD) is a common complication. Fistula formation is reported in up to 50% of the patients and a significant proportion of them present intra-abdominal fistulas, a very invasive and challenging complication for the surgeon (1). In a cohort of 213 CD patients, a frequency of 26% cases with intra-abdominal fistulas was reported and Yoon and coworker identified a similar percentage of cases, 34% (2, 3).

Studies focused on CD patients with this complication are mainly devoted to examining their clinical presentation and treatment. Differences in clinical and pathologic characteristics of patients with CD with and without fistulas seem not extensive but patients with intra-abdominal fistulas have more frequent other CD-related sepsis, such as intra-abdominal abscess or perianal abscess and fistula (3).

It has been suggested that the driving force of fistulas formation is the epithelial-to-mesenchymal transition (EMT): intestinal epithelial cells acquire the ability to penetrate into the deeper layers of the gastrointestinal wall, causing tissue damage and the formation of strictures that connect the gut to other intestinal tracts or organs (4). This mechanism involves the activation of matrix remodeling enzymes, such as the matrix metalloproteinases MMP-3 and MMP-9. MMP-9 in particular, is a gelatinase abundantly expressed around CD fistulas and found in granulocytes, mononuclear cells and fibroblasts suggesting an important role in fistula development (5). Goffin and coworkers reported that MMP-9 degradation products were significantly higher in the serum of patients with penetrating CD and that selective anti-MMP-9 monoclonal antibodies reduced collagen deposition in a heterotopic xenograft mouse model of intestinal fibrosis (4).

MiR-126 is an endothelial-enriched miRNA that prevents intestinal mucosal barrier dysfunction and acts as an inhibitor of NF-κB signaling pathway (6, 7). Interestingly, Ye and coworkers demonstrated that VEGF and MMP9 expression was induced by hypoxic conditions and that treatment with miR-126 mimics down-regulated both genes, in vessel endothelial cells (8).

The aim of the present work was to correlate the occurrence of intestinal fistulization to the clinico-pathological features of CD patients and to explore the involvement of miR-126 and MMP9 in this manifestation.

## Methods

### Patient Enrollment and Sample Collection

A cohort of 31 patients with Crohn’s disease with complicated disease requiring surgery, were consecutive enrolled at the Digestive Surgery Unit of the Careggi Hospital. Samples of affected mucosa were harvested at surgery, fixed in Carnoy’s solution and embedded in paraffin.

Blood samples were collected in EDTA-coated tubes, centrifuged at 2,000 rpm for 10 min to isolate plasma and stored at −20°C, until analysis.

Demographic and clinical data (age, gender, smoking habit, familial IBD, disease duration, disease location, the interval between diagnosis and first surgery, number of surgeries, disease behavior, presences of abdominal fistulae and/or abscesses, short-term post-operative complications and length of hospital stay) were retrieved from medical records. Disease activity was identified according to the CD activity index (CDAI) and endoscopic disease severity was assessed using the Rutgeert’s score (i0-i4).

### Determination of Circulating miR-126

Total RNA was extracted from 200 µL of plasma by using TRIzol (Invitrogen, Life Technologies, Carlsbad, CA, USA), according to the manufacturer instructions. For plasma miR-126 determination, the Qiagen miRCURY® LNA®miRNA PCR System was used: miRCURY LNA RT Kit for miRNA-specific cDNA synthesis and the miRCURY LNA SYBR Green PCR Kit and miRCURY LNA miRNA PCR Assay for quantitative real-time PCR amplifications. The analyses were performed using the Rotor-Gene Q thermal cycler (Qiagen, Hilden, Germany) and 40 cycles at 9°C for 15 s, 55°C for 30 s and 70°C for 30 s. RNU6B was used as endogenous control and relative expressions were calculated as 2^−ΔCt^ values.

### Immunohistochemistry

The immunohistochemistry determination of MMP9 protein was performed on histological slices (4 µm tick); primary antibodies: purified mouse monoclonal anti MMP9 (1:100; ThermoFisher Scientific, USA); secondary stained was performed by using the Vectastain Elite ABC universal kit (Vector Laboratories, USA) according to manufacturer’s specifications. Microscopic images were evaluated with the ACT-2U software program (Nikon, Instruments Europe, Badhoevedorp, Netherlands) connected via a camera to the microscope (Optiphot-2; Nikon).

## Elisa

MMP9 levels were measured in plasma samples using the QuickDetect™ MMP-9, human (BioVision Inc. CA, USA) Cayman Chemical, MI, USA) according to the manufacturer’s specifications, and expressed as ng/mL of plasma.

### Data Analysis

Categorical variables were expressed as absolute numbers and percentages. Kolmogorov–Smirnov test was used to assess normality distribution and continuous variables were reported as mean ± SEM if normally distributed or as median and interquartile range (IQR) if not. Comparison of continuous variables were performed by *t*-test or Mann-Whitney test (when data were non-normally distributed). Differences between proportions were assessed using the Fisher exact test. *P* values <0.05 were considered significant. The diagnostic accuracy of circulating MMP9 for identifying intra-abdominal fistulae cases was tested by Receiver Operating Characteristic (ROC) curve calculation.

To identify factors affecting plasma MMP9 levels, a stepwise multiple regression with backward selection was performed, taking into account the following factors as independent variables: age, gender, disease duration, circulating miR-126 levels, CDAI, disease location and behavior, surgical and endoscopic (Rutgeert’s score) recurrence and presence of intra-abdominal fistula.

Statistical analysis were performed using SPSS Statistics statistical software (version 27, SPSS Inc., Chicago, IL, USA) and GraphPad Prism 7.

## Results

Thirty-one CD patients with a mean age of 55.8 ± 2.7 years and a mean disease duration of 16.87 ± 1.95 years were enrolled at surgery. About 55% of the cases were surgical recurrences. Nine patients presented intra-abdominal fistulas (29%), 6 entero-enteric and 3 entero-cutaneous.

By comparing cases with and without intra-abdominal fistulas, we noted that the first group exhibited a significantly higher CDAI (*p* < 0.0001), Table 1.

**Table 1:** Demographic and clinical characteristics of CD patients with or without intra-abdominal fistulas.

	With	Without	
*n*	*9*	*22*	* *
Age	58 (51–63)	54 (43.5–71)	NS
Gender, M	3 (33.33%)	13 (59.09%)	NS
Smokers or former smokers	2 (22.22%)	10 (45.45%)	NS
Disease duration	16.44 ± 3.66	17.05 ± 2.36	NS
Disease location, ileum	5 (55.56%)	16 (72.73%)	NS
Δ diagnosis-surgery (years)	2 (1–10.5)	2.5 (0–4.25)	NS
CDAI	350 (300–350)	250 (250–300)	<0.0001
Post-operative days	7 (6.5–9)	6 (6–8)	NS
Clavien-Dindo, ≥2	3 (33.3%)	4 (18.2%)	NS
Surgical recurrence, yes	5 (55.56%)	12 (54.54%)	NS
Rutgeert’s score ≥i2	4 (44.44%)	4 (18.18%)	NS

*Data are median (IQR), mean ± SE or number (percentage).*

**Figure 1:**
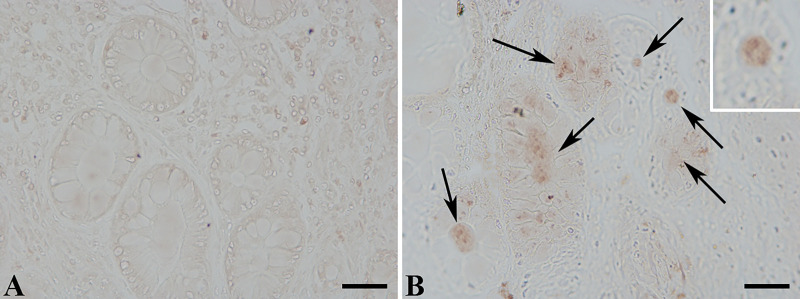
MMP9 protein expression determined by immunohistochemistry with an anti-MMP9 antibody (brown signal). (**A**) Histological section from a Crohn’s patient without fistulas; (**B**) section harvested from a patient with intra-abdominal fistula. The arrows pointed out the presence of MMP9 staining in the crypt lumen and in colonocytes.

In the plasma of CD patients with intra-abdominal fistulas we found a significantly higher level of circulating MMP9: 1,604 (1,488–2,222) vs. 761.6 (625.7–950.6) ng/mL compared to patients without internal fistulas, median (interquartile range, IQR), *p* < 0.0001. Peripheral MMP9 levels were correlated with the presence of MMP9 protein in sections of inflamed mucosa, located both intracellularly and in the lumen of intestinal crypts, as pointed out by the immunohistochemistry evaluation Figure 1.

ROC curve analysis calculated an AUC value of 0.9798, a specificity of 95.45 (ability to correctly identify true negative) and a sensitivity of 88.89 (ability to correctly identify true positive), *p* < 0.0001, with a threshold value >1,445 ng/mL of plasma (Likelihood ratio of 19.56).

**Figure 2:**
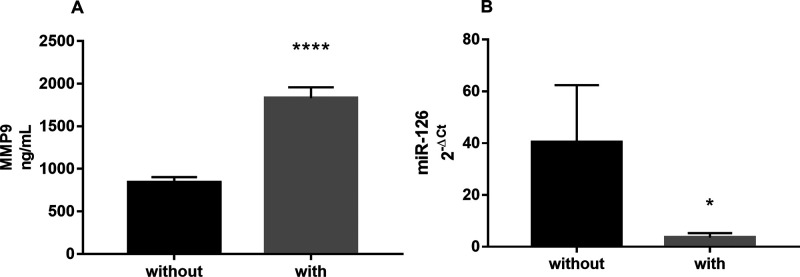
(**A**) Circulating levels of MMP9 and (**B**) miR-126, measured in the plasma of CD patients with (*n* = 9) or without intra-abdominal fistulas (*n* = 22). Data are expressed as mean ± SE **** *p* < 0.0001 and *p < 0.05 by Mann-Whitney test.

As shown in Figure 2, circulating miR-126 was significantly lower in the plasma of CD patients with intra-abdominal fistulas compared with patients without fistulas [0.6 (0.3–6.7) vs. 12.29 (1.75–26.8), median (IQR); *p* = 0.0231].

**Figure 3:**
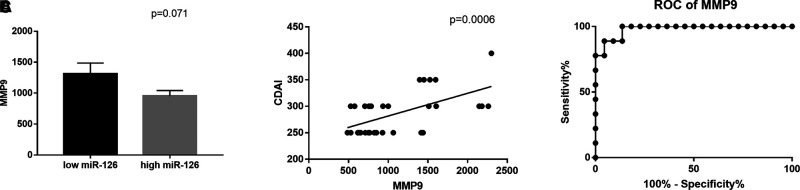
(**A**) Correlation between circulating MMP9 and miR-126 expression and (**B**) with CDAI scores. (**C**) Receiver operating characteristics (ROC) curve for the ability of circulating MMP9 to discriminate between CD patients with or without intra-abdominal fistulas: Area Under Curve (AUC) 0.9798, sensitivity 88.89 and specificity 95.45, *p* < 0.0001.

MMP9 levels were positively correlated with the CDAI score and showed a borderline, inverse correlation with circulating miR-126 expression (*p* = 0.07), Figure 3.

By multiple regression analysis, we observed that the presence of intra-abdominal fistulas (*p* < 0.0001) and Rutgeert’s score (*p* < 0.0170) were the only independent factors correlated with circulating MMP9 levels.

## Discussion

In a significant percentage of Crohn’s patients, disease progression is characterized by intestinal complications such as strictures and fistulas. The development of disease-related complications, affects the quality of life of these patients, may require surgical treatment and increases healthcare costs.

Depending on the severity and on the fistula site, different medical and surgical strategies are possible and either symptomatic or not, internal fistulas always represent a risk of abscess formation.

The first line therapy is represented by antibiotics and, apart from azathioprine or 6-mercaptopurine and anti-TNF mAbs, no other therapeutics are available to treat fistulas. Overall, since pharmacological options and surgery are only partially effective and about one-third of patients suffer from recurring fistulas (9), further research is needed to better understand the pathogenesis of intra-abdominal fistulas, and to develop novel pharmacological options and new strategies for monitoring fistulas formation in CD patients.

MMP9 is predominantly upregulated in animal models of colitis and plays a major role in driving gut inflammation in both infectious and chemical-induced colitis. Transgenic mice over-expressing MMP9 had defective barrier function, altered mucin secretion and increased IL-8, and exhibited worsening of acute colitis in DSS or *Salmonella*-induced colitis models (10).

MMPs are involved in the typical lesions that characterize Crohn’s disease, transmural inflammation, fibrosis, and fistula formation. Many studies have shown that MMPs levels are correlated with disease activity and increased MMP-9 degradation products have been reported in serum and urine samples of IBD patients (4, 11, 12). Moreover, in CD patients with quiescent disease, higher levels of baseline MMP9 were associated to an increased risk for clinical flare (13). Shamseya and coworkers reported also that in both ulcerative colitis and CD patients, there were lower serum levels of MMP-9 in patients receiving biologics compared to those receiving conventional treatments (12). These data suggest that serum MMP-9 values might not only predict further relapse but also help to assess the pharmacological response.

Our ROC analysis results also indicated that the measurement of circulating levels of MMP9 can discriminate between CD patients with and without intra-abdominal fistulas. Since asymptomatic intra-abdominal fistulas are common, the determination of circulating MMP9 could be a useful biomarker for the evaluation of the risk of intestinal fistulization. Moreover, we observed that MMP9 is upregulated not only in intestinal fistulas with acute inflammation, as previously reported, but also in sections of inflamed mucosa of patients with internal fistulas suggesting a wide upregulation of this gelatinase in the gut of patients suffering for this complication and a high risk of fistula formation in other intestinal segments.

*In silico* target prediction analysis (TargetScan and DIANA tools) identified as potential target for miR-126-5p, several MMPs such as MMP2, 3, 13, 16, 10 and 20, but not MMP9. However, a number of studies suggested that miR-126 might indirectly regulate MMP9. In the study by Ye and coworkers (2014), VEGF and MMP9 were down-regulated after incubation with miR-126 mimics under hypoxic conditions while miR-126 inhibition slightly increased their expression (8). Similarly, Shi and coworkers observed that the activities of MMP9 decreased in response to miR-126-5p agomir in a model of aortic smooth muscle cells transition (14). MiR-126 and MMP9 have been also suggested to play a role in development of endometriosis and MMP9 in particular, seems to be a potential marker of the disease (15–17). It has been also proposed that miR-126 acts as a tumor suppressor inhibiting proliferation, migration, and invasion by suppressing MMP2 and MMP9 expression and inactivating JAK2/STAT3 signaling pathway through targeting the EMT transcription factor ZEB1 (18).

The role of MMPs in the formation of intra-abdominal fistulas suggested the development of compounds specifically designed to suppress their activity such as an anti-MMP9 mAb that was successfully tested in an experimental model in mice (4). In supernatants of homogenates from Crohn’s disease fistulas, Efsen and coworkers demonstrated that ramiprilate, the active metabolite of ramipril, inhibited MMP9 activity, dose-dependently. This interesting finding suggests to specifically design studies aimed at exploring if the prevalence of intra-abdominal fistulas is lower in hypertensive CD patient treated with this ACE-inhibitor.

The potential involvement of miR-126 in the pathophysiology of intra-abdominal fistula formation could also suggest the use of this miRNA for identifying new therapeutic strategies and /or for CD patients monitoring. In fact, it has been recently reported that serum miR-126 levels were associated to therapeutic response, being decreased after anti-TNF or glucocorticoids therapies, only in treatment responders (19).

## Conclusions

We performed a small, pilot study and to validate circulating MMP9 as biomarker for early diagnosis and/or clinical monitoring of CD patients with abdominal fistula, a larger, prospective study is needed. However, our preliminary report offers a number of interesting insights: despite the limited number of cases with intra-abdominal fistulas, we were able to identify differences associated to the presence of internal fistulas and significant correlations among circulating biomarkers and well-known markers of disease activity such as CDAI and Rutgeert’s score.

## Data Availability

The raw data supporting the conclusions of this article will be made available by the authors, without undue reservation.
